# Exploring the Impact of User Experience on Value Co-Creation Citizenship Behaviors in Virtual Brand Communities

**DOI:** 10.3390/bs16050768

**Published:** 2026-05-14

**Authors:** Jielin Yin, Yi Chang, Zhenzhong Ma, Yangyang Zhao, Jiaxin Qi

**Affiliations:** 1 Business School, Beijing Information Science & Technology University, Beijing 102206, China; yinjielin0924@bistu.edu.cn (J.Y.); 2024020835@bistu.edu.cn (Y.C.); 2023020778@bistu.edu.cn (Y.Z.); qjx2724@163.com (J.Q.); 2 School of Economics and Management, Harbin Institute of Technology, Shenzhen 518055, China; 3 Odette School of Business, University of Windsor, Windsor, ON N9B 3P4, Canada

**Keywords:** virtual brand community, user experience, relationship quality, value co-creation citizenship behavior, community support

## Abstract

With the proliferation of digital platforms, virtual brand communities have become important contexts for examining how individual perceptions shape discretionary behaviors in online environments. However, the mechanisms through which user experience translates into value co-creation behaviors remain underexplored. Drawing on relationship marketing theory and a behavioral perspective, this study develops and tests a theoretical model linking user experience to value co-creation citizenship behaviors through distinct dimensions of quality of relationship-satisfaction, trust, and commitment. Using a two-wave survey with 549 matched responses, we employ multiple regressions and bootstrapping analyses to assess mediation and moderation effects. The findings indicate that different dimensions of user experience have differential impacts on satisfaction, trust, and commitment, which in turn promote value co-creation citizenship behaviors, supporting their roles as central psychological mechanisms. Specifically, affective and behavioral experiences exert significant positive impacts on value co-creation citizenship behaviors, mediated by all three dimensions (satisfaction, trust, and commitment), whereas the influences of sensory and intellectual experiences are only mediated by two dimensions (satisfaction and trust) of the quality of relationship. In addition, perceived community support strengthens the relationship between satisfaction and value co-creation citizenship behaviors, while it exerts no significant moderating effects on the impact of trust or commitment on value co-creation citizenship behaviors. By situating value co-creation within a behavioral framework, this study contributes to the literature by exploring the mechanism through which user experience influences voluntary, citizenship-like behaviors in digital communities from a relational perspective, and by identifying boundary conditions under which these effects are amplified.

## 1. Introduction

Along with the rapid expansion of digital economy, virtual brand communities (VBCs) have emerged as critical contexts in which user–firm interactions are enacted and value is co-created through ongoing social exchanges (N. Wang et al., 2021, 2022, 2023; Zhang et al., 2021). By transcending temporal and spatial constraints, VBCs emphasize interactivity, participation, and user agency, enabling individuals to shift from passive recipients of brand messages to active contributors to value creation (Healy & McDonagh, 2013; Zheng et al., 2023) and strengthening their emotional attachment and brand identification (Z. Wang & Yang, 2025). This transformation reflects a broader service-dominant logic, which conceptualizes value as emerging from the integration of resources and relational processes rather than being embedded solely in products (Vargo & Lusch, 2014). Consequently, VBCs are not merely communication channels but dynamic social systems in which behavioral engagement and relational dynamics jointly shape value creation outcomes (Schau et al., 2009).

Within these systems, community users’ discretionary contributions, such as providing feedback, assisting other members, and advocating for the brand, play a pivotal role in sustaining community vitality and enhancing firms’ adaptive capacity in competitive markets (Frasquet-Deltoro et al., 2019; Healy & McDonagh, 2013). These discretionary behaviors, often conceptualized as value co-creation citizenship behaviors (VCCBs), extend beyond users’ immediate self-interest and reflect voluntary, prosocial engagement within the communities (Yi & Gong, 2013). Prior research distinguishes between participation behaviors, which are task-oriented and self-serving, and citizenship behaviors, which are other-oriented and community-enhancing (Yi & Gong, 2013). Empirical evidence suggests that VCCBs are particularly consequential because they increase community participation, improve information diffusion, and enhance the sustainability of VBCs (S. Yang et al., 2023). Accordingly, understanding the antecedents of VCCBs has become a central concern in research on digital communities and value co-creation. However, despite this growing interest, there remains limited understanding of how user experience translates into discretionary citizenship behaviors through relational processes in VBCs.

Existing studies have identified a range of drivers of VCCBs, broadly falling into two categories: individual-level factors and contextual influences. At the individual level, constructs such as affective commitment and positive emotional interaction have been shown to stimulate users’ willingness to contribute beyond their formal roles (Curth et al., 2014; Jung & Yoo, 2017). At the contextual level, features of the community environment, including perceived support and social climate, have been found to shape user behaviors (Gao et al., 2023; D. H. Zhu et al., 2016). While these studies provide valuable insights, they remain fragmented and offer limited understanding of how core experiential perceptions are translated into sustained, discretionary engagement. In particular, insufficient attention has been given to the internal psychological mechanisms through which user experience influences VCCBs, as well as to the boundary conditions under which such effects are strengthened or attenuated.

User experience in virtual communities refers to individuals’ cognitive and affective responses to their interactions within a digital environment, and it represents a foundational determinant of user behavior in VBCs (Schmitt, 1999; Verhoef et al., 2009). With the rapid advancement of artificial intelligence technologies, firms are increasingly able to design and deliver personalized, adaptive experiences through intelligent recommendation systems and interactive interfaces, thereby shaping users’ perceptions and behavioral tendencies in more nuanced ways (Ameen et al., 2021; Marti et al., 2024). Positive user experience can enhance users’ favorable evaluations of both the brand and the community, potentially motivating them to engage in VCCBs (Bansal & Thakur, 2024; Le et al., 2024). However, the mechanisms underlying this relationship remain contested.

While existing research has examined user experience and its outcomes in online communities, several important research gaps remain. First, existing studies have often treated user experience as a unidimensional construct or have focused on its overall effect, overlooking the possibility that different experiential dimensions may exert distinct influences on user perceptions and behaviors (Brakus et al., 2009; Lemon & Verhoef, 2016). As a result, the nuanced mechanisms through which specific types of experience shape user outcomes remain unexplored. Second, much prior research has primarily focused on the direct effects of user experience on behavioral outcomes such as participation, engagement, or co-creation behaviors, and relatively limited attention has been given to the underlying relational processes that connect experience to behavior (Verhoef et al., 2009; Hollebeek et al., 2014). Third, while relationship quality has been widely recognized as an important construct, many studies have treated it as a higher-order or aggregated concept, without systematically examining the differentiated roles of its core dimensions, including satisfaction, trust, and commitment (Morgan & Hunt, 1994; Palmatier et al., 2006). This limits our understanding of how distinct relational states may operate as separate mechanisms linking experience to behavior. Finally, although contextual factors are known to shape user behavior in online environments, relatively little research has examined how community-level support influences the translation of user experience into voluntary, extra-role contributions (Y. Wang & Hajli, 2014; D. H. Zhu et al., 2016).

To address these gaps, this study develops a theoretical framework grounded in relationship marketing theory to explain how user experience influences VCCBs through three dimensions of the quality of relationship, while moderated by community support. Relationship marketing theory posits that behavioral outcomes are not driven directly by stimuli but are mediated through the development of relational bonds characterized by satisfaction, trust, and commitment (Morgan & Hunt, 1994; Palmatier et al., 2006). These relational constructs capture the cumulative cognitive and affective evaluations that arise from repeated interactions and serve as key mechanisms linking perceptions to behavior. Moreover, this theory emphasizes the importance of contextual factors, such as relational climate and support structures, in shaping the effectiveness of these relational bonds and amplifying their behavioral consequences (Palmatier et al., 2006).

In the context of VBCs, the quality of relationship represents a critical linkage between users and the community, reflecting the extent to which users perceive the community as trustworthy, satisfying, and worthy of continued engagement. High-quality relationships not only enhance users’ consumption and advocacy behaviors but also motivate them to engage in discretionary contributions that sustain the community ecosystem (C. F. Chen & Myagmarsuren, 2011; Chi et al., 2020; Jang et al., 2008; Ulaga & Eggert, 2006). At the same time, community support, defined as users’ perceptions of the extent to which the community provides assistance, resources, and encouragement, serves as an important boundary condition that strengthens the translation of relational evaluations into citizenship behaviors. By incorporating both mediating and moderating mechanisms, this study provides a more comprehensive account of how experiential perceptions are internalized and enacted for VCCBs within digital communities.

Taken together, this research makes several contributions. First, it adds to the literature on value co-creation by unpacking the behavioral pathway through which user experience influences VCCBs, moving beyond direct-effect models to highlight the central roles of relational mechanisms. Second, it deepens the conceptualization of user experience by treating it as a multidimensional construct and examining its differentiated effects. Third, it extends relationship marketing theory into the context of virtual, technology-enabled communities, demonstrating how relational processes operate in digitally mediated environments. Finally, it contributes to a more nuanced understanding of when and how user experience translates into sustained, discretionary engagement by identifying community support as a key boundary condition. Through this integrative approach, our study provides a theoretically grounded and behaviorally informed explanation of value co-creation behaviours in VBCs.

## 2. Theoretical Background

### 2.1. Relationship Marketing Theory

Relationship marketing theory (RMT), originally proposed by Berry (1983), emphasizes the importance of establishing, maintaining, and enhancing long-term relationships between firms and customers to achieve sustained value creation. In contrast to transactional marketing, which focuses on short-term exchanges, RMT highlights relational processes, such as trust, commitment, and emotional bonding, as key drivers of enduring behavioral engagement (Morgan & Hunt, 1994). Within this perspective, customers are not passive recipients of value but active participants in collaborative value co-creation processes (Du et al., 2022; Gummesson, 2002; Yin et al., 2025).

As the theory has evolved, the quality of relationship has emerged as a central construct for assessing the strength and effectiveness of firm–customer relationships (Hennig-Thurau et al., 2002; Palmatier et al., 2006). In this study, the relationship is conceptualized as a user–community rather than traditional firm–customer relationship. The commitment–trust framework posits that the quality of relationship reflects customers’ overall evaluation of relational exchanges and is typically operationalized through satisfaction, trust, and commitment (Morgan & Hunt, 1994). Within VBC contexts, the dimensions of satisfaction, trust, and commitment of relationship are particularly relevant as they capture distinct yet complementary relational states (Chiu et al., 2015). Satisfaction reflects users’ evaluative responses to their experiences within the community, trust represents confidence in the reliability and integrity of the community environment, and commitment captures the intention to maintain a long-term relationship with the community. Together, these dimensions provide a comprehensive representation of user–community relational quality in VBCs. High levels of quality indicate that customers are satisfied with interactions, trust the focal entity, and are willing to maintain the relationship over time. As a relational asset, high-quality relationships reduce uncertainty, lower transaction costs, and enhance cooperative behaviors and positive behavioral responses (Palmatier et al., 2006; Tajvidi et al., 2021).

Compared with alternative perspectives such as social exchange theory and social identity theory (Delgado-Ballester & Fernandez Sabiote, 2015; Hasan et al., 2024; Y. Lin & Choe, 2022; PhamThi & Ho, 2024; M. Yang et al., 2024), which often focus on cost–benefit evaluations or identity-based motivations, RMT provides a more process-oriented explanation by emphasizing how relational bonds are formed and strengthened through ongoing interactions, thereby linking experiential perceptions to sustained behavioral engagement. With the rise of digital platforms and the platform economy, RMT has been increasingly extended to VBCs, where user–firm relationships are mediated through technology-enabled interactions (Lamberton & Stephen, 2016; Riley & Nicewicz-Scott, 2025). In these environments, experiential interactions serve as key antecedents of relational evaluations, as users continuously form perceptions through engagement with community content and members (Schmitt, 1999; Verhoef et al., 2009). High-quality relationships in VBCs not only strengthen user loyalty but also stimulate VCCBs, such as helping others, providing feedback, and advocating for the brand (Yi & Gong, 2013). Moreover, RMT underscores the role of contextual factors in shaping relational processes, suggesting that supportive environments and resource availability enhance the formation of high-quality relationships and amplify its behavioral consequences (Palmatier et al., 2006). In VBCs, community support, manifested through governance mechanisms, technical infrastructure, and resource provision, constitutes a critical contextual condition that can strengthen relational bonds and facilitate their translation into citizenship behaviors (Liu et al., 2020; D. H. Zhu et al., 2016).

### 2.2. User Experience in Virtual Brand Communities

The concept of user experience originates from Schmitt (1999), who conceptualizes it as a subjective response arising from the interaction between cognitive and affective processes triggered by external stimuli. Extending this notion to digital contexts, user experience in VBCs reflects individuals’ holistic perceptions formed through ongoing interactions, encompassing perception, cognition, and behavioral engagement (Z. Li et al., 2019; Mahmoud et al., 2018; Tian et al., 2010). Building on this literature, this study defines user experience in VBCs as a comprehensive subjective evaluation shaped by users’ continuous interaction and communication within VBCs.

Prior research has conceptualized user experience as a multidimensional construct. Among these, the four-dimensional framework proposed by Brakus et al. (2009) is widely adopted and theoretically robust. In this study, we define user experience as a multi-dimensional structure, consisting of four dimensions—sensory, affective, intellectual and behavioral—rather than a unified higher-order construct. Sensory experience refers to users’ multisensory perceptions (e.g., visual and auditory stimuli) arising from their interaction with the virtual brand community environment. Affective experience captures the emotional responses generated through social interaction, communication, and engagement with other community members. Intellectual experience reflects the extent to which users are cognitively stimulated through information exchange, problem solving, and knowledge sharing within the community. Behavioral experience refers to the extent to which participation in community activities encourages users’ active engagement, such as contributing content, interacting with others, and taking part in community-driven initiatives.

By adopting this multidimensional perspective, the study captures the heterogeneity of experiential stimuli embedded in community participation and provides a more fine-grained understanding of how different experiential components shape subsequent relational and behavioral outcomes in VBCs.

### 2.3. Value Co-Creation Citizenship Behavior

The concept of value co-creation citizenship behavior (VCCB) is rooted in organizational citizenship behavior (OCB), originally introduced by Organ (1988) to describe voluntary, extra-role behaviors that contribute to organizational effectiveness. Extending this concept to the consumer domain, user citizenship behavior refers to voluntary and proactive actions performed beyond formal role expectations, aimed at enhancing the value of products or services (Tang & Jiang, 2018). Within value co-creation contexts, such behaviors represent a higher-order form of user engagement, characterized by discretionary contributions that benefit the broad community (X. Yang & Tu, 2018). Importantly, VCCBs are conceptually distinct from routine participation behaviors, which are typically task-oriented and required for basic community functioning. Instead, VCCBs refer specifically to voluntary, extra-role, and community-enhancing actions that go beyond users’ immediate self-interest and formal participation expectations (Yi & Gong, 2013).

VCCBs typically include behaviors such as helping other users, providing suggestions, offering recommendations, and demonstrating tolerance toward service failures or community imperfections. These behaviors exhibit three defining characteristics: they are value-creating, voluntary, and extra-role in nature (Tang & Jiang, 2018; X. Yang & Tu, 2018; Yi & Gong, 2013). In VBCs, where value emerges through collective participation and interaction, VCCBs play a critical role in sustaining community vitality and enhancing collective outcomes. Accordingly, this study defines VCCBs as users’ voluntary and self-initiated behaviors within VBCs that generate additional value and contribute to the effective functioning of the community.

### 2.4. Quality of Relationship

The quality of relationship refers to the overall assessment of the strength and positivity of a relationship, reflecting the extent to which relational expectations and needs are fulfilled (Holmlund, 2001). In digital and service contexts, high-quality relationships have been conceptualized as integrative evaluations encompassing trust, satisfaction, and relational continuity (Chang et al., 2015; Xin & Wang, 2018). In VBCs, high-quality relationships capture the perceived strength of the connection between users and the community, reflecting both the quality of interactions and the stability of the relationship.

Although no universal consensus exists regarding its dimensionality, satisfaction, trust, and commitment are consistently identified as the core components of high-quality relationships (Chumpitaz Caceres & Paparoidamis, 2007; Dorai et al., 2021; Itani et al., 2019). Satisfaction reflects users’ positive evaluations of their experiences when perceived outcomes meet or exceed expectations (Schmitt, 1999). Trust denotes users’ confidence in the community’s reliability, integrity, and benevolence, including the belief that the community will act in users’ interests and avoid opportunistic behavior (C. W. Lin et al., 2019). Commitment represents users’ emotional attachment and willingness to maintain a long-term relationship with the community, reflecting a deeper level of relational engagement (X. W. Wang et al., 2019).

Drawing on the above literature, this study contends that the quality of relationship consists of three dimensions, satisfaction, trust, and commitment, which are key dimensions of a relationship that operate in parallel to relate user experience to value co-creation citizenship behaviors. These three relational dimensions serve as key psychological mechanisms through which experiential perceptions are internalized and translated into behavioral outcomes.

### 2.5. Community Support

The concept of community support is derived from organizational support theory, which posits that individuals develop general beliefs regarding the extent to which an organization values their contributions and cares about their well-being (Eisenberger et al., 1986). Extending this concept to digital communities, perceived community support reflects users’ evaluations of the extent to which the community provides resources, assistance, and emotional care (J. Fan & Lin, 2020; Meng, 2017).

In VBCs, community support encompasses both instrumental and emotional dimensions, including information sharing, technical assistance, social recognition, and mutual help among members. Through these mechanisms, community support enhances users’ sense of belonging and strengthens their engagement with the community. Moreover, by providing enabling conditions for interaction and participation, community support facilitates resource exchange and reinforces relational bonds (Yu et al., 2020).

Accordingly, this study defines community support as the set of structural and relational resources provided within VBCs, such as information sharing, emotional care, and incentive mechanisms, that enhance users’ perceived support and participation willingness. From a relational perspective, community support not only contributes to the formation of high-quality relationships but also functions as a boundary condition that strengthens the translation of relational evaluations into value co-creation citizenship behaviors through the acquisition of structural and emotional resources.

## 3. Hypotheses

### 3.1. User Experience and the Quality of Relationship

Within VBCs, user experience constitutes a primary stimulus that shapes users’ relational evaluations. From a relationship marketing perspective, experiential interactions serve as the foundation upon which satisfaction, trust, and commitment are developed through repeated exchanges (Morgan & Hunt, 1994; Palmatier et al., 2006). Specifically, different user experiences including sensory, affective, intellectual, and behavioral capture distinct cognitive and affective responses that may exert differentiated effects on satisfaction, trust, and commitment as distinct dimensions of the quality of relationship.

First, satisfaction is a post-evaluative judgment derived from users’ comparison between expected and actual experiences (L. Deng et al., 2010). This evaluative process is grounded in Expectation–Confirmation theory, which suggests that satisfaction arises when perceived performance meets or exceeds prior expectations, thereby linking experiential outcomes to cognitive confirmation mechanisms (Bhattacherjee, 2001). Sensory experience enhances perceptual clarity and immersion, thereby improving users’ evaluative judgments of the community (L. Wang & Huang, 2023). Specifically, by improving interface/front end presentation and environmental cues, sensory experience facilitates smoother information processing, which in turn strengthens users’ overall evaluative efficiency and perception of platform quality (Eroglu et al., 2001; Rose et al., 2012). Affective experience strengthens emotional resonance, which is a key determinant of satisfaction in interactive environments (W. Wang et al., 2024). Emotional responses/Positive emotions such as enjoyment and pleasure directly influence users’ affective evaluations, and prior studies suggest that such affective reactions serve as immediate antecedents of satisfaction, particularly in socially interactive and experience-rich contexts (Hollebeek, 2011; Brodie et al., 2013). Intellectual experience, through knowledge acquisition and cognitive stimulation, further reinforces users’ perceived utility and fulfillment (Qian et al., 2023). From a cognitive appraisal perspective, intellectual stimulation enhances perceived usefulness and value, thereby contributing to satisfaction through rational evaluation processes (Bhattacherjee, 2001). Behavioral experience, reflected in effective interaction and feedback exchange, enhances perceived responsiveness and reinforces satisfaction. Active participation and interactive feedback increase users’ sense of control and perceived interactivity. Behavioral engagements further strengthen satisfaction outcomes through value co-creation mechanisms (Vargo & Lusch, 2008).

**H1a.**  *Sensory experience positively influences satisfaction.*

**H1b.**  *Affective experience positively influences satisfaction.*

**H1c.**  *Intellectual experience positively influences satisfaction.*

**H1d.**  *Behavioral experience positively influences satisfaction.*

Second, trust reflects users’ confidence in the reliability and integrity of the community and is shaped by experiential cues that reduce uncertainty and perceived risk (Martin et al., 2015). From a theoretical perspective, particularly in online environments where information asymmetry and lack of physical/experiential cues increase perceived risk (Gefen et al., 2003), sensory experience increases transparency and information richness, which enhances users’ confidence in the community (Shamim & Mohsin Butt, 2013). Specifically, clear interface design, vivid presentation, and accessible information structures improve perceived transparency and reduce ambiguity, thereby lowering uncertainty and facilitating trust formation (Cyr et al., 2009; Eroglu et al., 2001). Affective experience fosters social interaction and emotional bonding, thereby strengthening interpersonal trust. Emotional connections developed through positive interactions can generate feelings of familiarity and psychological closeness, which are critical foundations of trust, especially in virtual communities where relational cues are otherwise limited (Hassanein & Head, 2007; McAllister, 1995). Intellectual experience, through deeper cognitive engagement and knowledge sharing, promotes users’ identification with the community, which underpins trust formation (L. Wang & Xia, 2023). Through cognitive engagement, users are more likely to perceive the community as competent and valuable, reinforcing beliefs in its expertise and integrity, which are central dimensions of trust (DeLone & McLean, 2003). Behavioral experience, particularly through useful information exchange and interactive feedback, also reinforces perceived usefulness and reliability, thereby enhancing trust.

**H1e.**  *Sensory experience positively influences trust.*

**H1f.**  *Affective experience positively influences trust.*

**H1g.**  *Intellectual experience positively influences trust.*

**H1h.**  *Behavioral experience positively influences trust.*

Third, commitment represents a relational state reflecting users’ emotional attachment and willingness to maintain a long-term relationship (Morgan & Hunt, 1994). Compared to satisfaction and trust, commitment is more strongly driven by affective and behavioral experiences that generate emotional resonance and relational embeddedness. Affective experience enhances users’ identification with and attachment to the community (G. Fan & Wu, 2019). Behavioral experience, through sustained interaction and reciprocal engagement, reinforces users’ sense of belonging and dependence on the community (Algesheimer et al., 2005). In contrast, sensory and intellectual experiences primarily stimulate short-term perceptual and cognitive evaluations rather than deep emotional attachment (Brakus et al., 2009) and thus are less likely to directly foster enduring commitment within VBCs. Therefore, it is hypothesized that only affective experience and behavioral experience, not sensory or intellectual experiences, significantly influence commitment.

**H1i.**  *Affective experience positively influences commitment.*

**H1j.**  *Behavioral experience positively influences commitment.*

### 3.2. Quality of Relationship and Value Co-Creation Citizenship Behavior

Relationship marketing theory posits that high-quality relationships motivate cooperative and discretionary behaviors that extend beyond formal obligations (Morgan & Hunt, 1994; Palmatier et al., 2006). In VBCs, the quality of relationship, captured by satisfaction, trust, and commitment, serves as a key driver of value co-creation citizenship behaviors (VCCBs), which are voluntary and community-enhancing in nature.

Satisfaction increases users’ willingness to reciprocate positive experiences through active participation and contribution. Trust reduces perceived risk and psychological resistance, thereby encouraging users to engage in cooperative and value-creating behaviors (Cao et al., 2023; See-To & Ho, 2014). Commitment, as a deeper relational bond, reflects users’ intrinsic motivation to sustain long-term engagement, which translates into consistent citizenship behaviors such as advocacy, helping, and knowledge sharing (Morgan & Hunt, 1994; Phua et al., 2017). Specifically, satisfaction reflects a positive evaluative judgment that motivates users to reciprocate favorable experiences through voluntary contributions, trust reduces perceived uncertainty and risk associated with engaging in value co-creation activities and commitment represents a durable psychological attachment that fosters identity-consistent and sustained citizenship behaviors.

**H2a.**  *Satisfaction positively influences VCCBs.*

**H2b.**  *Trust positively influences VCCBs.*

**H2c.**  *Commitment positively influences VCCBs.*

### 3.3. The Mediating Role of the Quality of Relationship

Relationship marketing theory further suggests that the effects of experiential stimuli on behavioral outcomes are primarily indirect, operating through relational mechanisms (Morgan & Hunt, 1994; Palmatier et al., 2006). In VBCs, user experience shapes relational evaluations (i.e., satisfaction, trust, and commitment), which in turn drive VCCBs.

In this study, we conceptualize the quality of relationship not as a single higher-order mediator, but as a set of distinct relational mechanisms. Satisfaction, trust, and commitment are treated as separate mediating pathways through which user experience influences value co-creation citizenship behavior. More specifically, positive experiences enhance satisfaction by fulfilling users’ expectations, which motivates reciprocal contributions to the community (Z. Deng et al., 2010; N. Wang et al., 2023). Similarly, experiential interactions facilitate trust formation by reducing uncertainty and fostering confidence in the community, thereby encouraging users to engage in value co-creation behaviors (Du et al., 2022; Gan & Wang, 2015; Tang & Jiang, 2018; Yin et al., 2025). Commitment emerges from sustained satisfaction and trust, representing a deeper psychological attachment that drives long-term and stable engagement in VCCBs (G. Fan & Wu, 2019; Gruen, 1995).

However, different dimensions of user experience may exert heterogeneous effects through distinct relational pathways. While satisfaction and trust are expected to mediate the effects of all experiential dimensions, commitment is more likely to mediate the effects of affective and behavioral experiences, which are closely associated with emotional attachment and relational embeddedness.

**H3a.**  *Satisfaction mediates the relationship between sensory experience and VCCBs.*

**H3b.**  *Satisfaction mediates the relationship between affective experience and VCCBs.*

**H3c.**  *Satisfaction mediates the relationship between intellectual experience and VCCBs.*

**H3d.**  *Satisfaction mediates the relationship between behavioral experience and VCCBs.*

**H3e.**  *Trust mediates the relationship between sensory experience and VCCBs.*

**H3f.**  *Trust mediates the relationship between affective experience and VCCBs.*

**H3g.**  *Trust mediates the relationship between intellectual experience and VCCBs.*

**H3h.**  *Trust mediates the relationship between behavioral experience and VCCBs.*

**H3i.**  *Commitment mediates the relationship between affective experience and VCCBs.*

**H3j.**  *Commitment mediates the relationship between behavioral experience and VCCBs.*

### 3.4. The Moderating Role of Community Support

Beyond relational mechanisms, relationship marketing theory emphasizes that contextual conditions shape the extent to which relational evaluations translate into behavioral outcomes (Palmatier et al., 2006). In VBCs, community support represents a critical contextual resource that enhances users’ perceived value and strengthens relational bonds.

Community support, manifested through emotional care, informational assistance, and technical infrastructure, enhances users’ sense of belonging and perceived reciprocity (L. Chen et al., 2022; J. Fan & Lin, 2020; Meng, 2017). From a social exchange theory perspective, such multidimensional support functions not only reduce users’ perceived uncertainty but also strengthen perceived relational obligation and belongingness within the community (Cropanzano & Mitchell, 2005). When users perceive high levels of support, they are more likely to reciprocate through voluntary contributions, consistent with the norm of reciprocity (Pang et al., 2022). Moreover, supportive environments reinforce users’ positive relational evaluations and amplify their behavioral consequences (Liu et al., 2020; D. H. Zhu et al., 2016).

Accordingly, community support is expected to strengthen the positive effects of satisfaction, trust, and commitment on VCCBs.

**H4a.**  *Community support positively moderates the relationship between satisfaction and VCCB.*

**H4b.**  *Community support positively moderates the relationship between trust and VCCB.*

**H4c.**  *Community support positively moderates the relationship between commitment and VCCB.*

**Figure 1:**
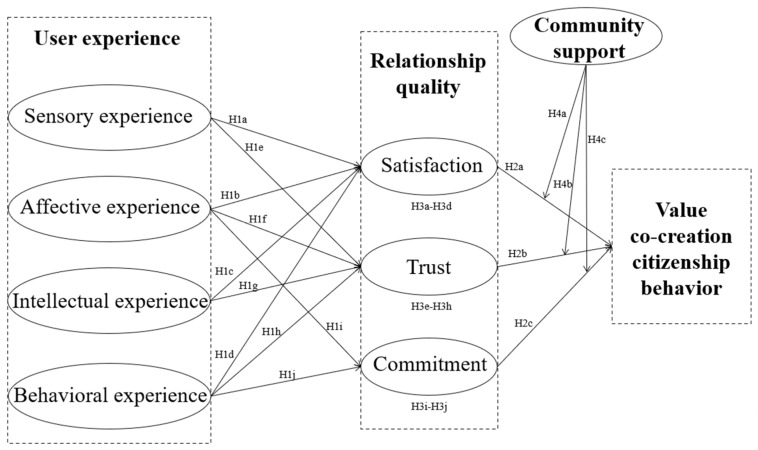
The proposed theoretical model.

Based on the above hypotheses, the proposed theoretical model of this study is depicted in Figure 1.

## 4. Methods

### 4.1. Sample and Procedure

To empirically test the proposed model, data were collected from users of several representative virtual brand communities (VBCs), including Xiaomi Community, VIVO Community, and NIO Community. These communities are all brand communities organized by related firms. These platforms were selected due to their high levels of user engagement and active participation in brand-related interactions. The survey was primarily distributed through community forums and user groups. Respondents were incentivized with the opportunity to receive a monetary reward ranging from 4 to 8 RMB after completing the questionnaire in order to enhance the response rate.

To reduce the risk of common method bias, a two-wave survey design was implemented (Podsakoff et al., 2003). This temporal separation between measurements helps mitigate common method bias by reducing respondents’ motivation to use the same cognitive frame when answering all items. Specifically, collecting independent and mediating variables in the first wave and dependent variables in the second wave introduces a time lag that decreases the likelihood of social desirability bias influencing responses to a certain extent. As a result, the observed relationships are less likely to be inflated by common method bias. Responses from the two waves were matched using the last four digits of respondents’ mobile phone numbers combined with their dates of birth, ensuring both anonymity and accurate pairing. This information was used solely for the purpose of matching responses across waves and was not used to identify individuals. All data were anonymized during analysis, and participants were informed that their responses would remain confidential and be used exclusively for academic research. Prior to the formal data collection, a pre-test was conducted with 50 users from the selected communities to assess the clarity, wording, and reliability of the measurement instruments. These pretest responses were used only for instrument refinement and were not included in the final sample. The results indicated that all constructs achieved satisfactory internal consistency, with Cronbach’s α coefficients exceeding the recommended threshold of 0.70.

The formal survey was conducted in two stages. In the first wave (15 August to 15 September 2024), data on the independent variables (user experience dimensions) and the mediating variables (satisfaction, trust, and commitment) were collected, yielding 703 valid responses. In the second wave (1 October to 1 November 2024), data on the moderating variable (community support) and the dependent variable (value co-creation citizenship behavior) were gathered from the same respondents. After removing incomplete and inconsistent responses and matching the two waves, a final sample of 549 valid paired questionnaires was obtained, corresponding to an effective response rate of 78.09%. Specifically, responses were excluded if they met one or more of the following criteria: (1) substantial missing data; (2) extremely short completion time (e.g., less than 2 min), indicating insufficient engagement; (3) logically inconsistent responses; or (4) failure to match responses across the two waves based on the identification information provided. T-test analyses were conducted to compare respondents who completed both waves and those who did not. No significant differences were found. The demographic characteristics of the sample are reported in Table 1.

### 4.2. Measures

All constructs were measured using established and validated scales from prior research, with minor wording adaptations to fit the context of virtual brand communities (VBCs). These adaptations mainly involved replacing generic terms such as “brand” or “company” with context-specific references to virtual brand communities and adjusting item wording to reflect users’ interactions within online community settings. The questionnaire was originally developed in English and translated into Chinese using a standard translation and back-translation procedure to ensure equivalence of meaning across languages. To ensure content validity and contextual appropriateness, the initial questionnaire was reviewed by three academic experts in marketing and two practitioners with extensive experience in managing VBCs. Based on their feedback, several items were refined to improve clarity and contextual relevance. The questionnaire began with an introductory statement explaining the study purpose, assuring respondents of anonymity and confidentiality, and emphasizing that there were no right or wrong answers and that responses would be used solely for academic research. All items were measured on a five-point Likert scale ranging from 1 (“strongly disagree”) to 5 (“strongly agree”). A five-point Likert scale was adopted to ensure ease of response and reduce respondent fatigue.

**User experience**: User experience was operationalized as a multidimensional construct and measured using the widely validated scale developed by Brakus et al. (2009). Consistent with prior research, the construct comprises four dimensions, sensory, affective, intellectual, and behavioral experiences, and includes a total of 12 items, with minor contextual adaptations to reflect interactions within VBCs.

**Quality of Relationship**: The dimension of the quality of relationship, which includes satisfaction, trust, and commitment, were measured differently. Satisfaction was measured using a three-item scale adapted from D. Li et al. (2006); trust was assessed with three items from Doney and Cannon (1997); and commitment was measured using three items adapted from Carlson et al. (2008).

**Community support**: Community support was measured using a four-item scale adapted from S. Wang et al. (2019), which refines prior measures of perceived support developed by Meng (2017) and Ye et al. (2015). These studies were conducted in online knowledge communities and brand community contexts, ensuring strong contextual alignment with VBCs. The items capture users’ perceptions of informational, emotional, and instrumental support within the community.

**Value co-creation citizenship behavior**: Value co-creation citizenship behavior (VCCB) was measured using a four-item scale adapted from Yi and Gong (2013), with modifications to reflect the specific context of VBCs. This scale was originally developed in a service environment. The scale captures users’ voluntary, extra-role behaviors, such as helping others, providing suggestions, and advocating for the brand.

The complete list of measurement items is provided in Table 2.

## 5. Data Analysis

### 5.1. Common Method Bias

We conducted Harman’s single-factor test to assess potential common method bias. The results showed that nine factors with eigenvalues greater than 1 explained 75.08% of the total variance. The first unrotated factor explained 27.61% of the variance, which is below the recommended threshold of 40%, indicating that common method variation is not a significant concern in this study. In addition, following the procedures recommended by Podsakoff et al. (2003). We added a common method factor in the CFA model and the result showed that the CFI, TLI and RMSEA values did not change much. Specifically, the CFI changed from 0.996 to 0.998, TLI changed from 0.995 to 0.996 and RMSEA changed from 0.013 to 0.012. This indicates that the fitting indices of the new model did not improve a lot. Thus, the common method bias does not seem to be a major problem in this study. To further assess the potential multicollinearity issue among variables, we calculated VIF (variance inflation factor) to check the potential problem of multicollinearity, and none of the VIFs was greater than 5, indicating that multicollinearity is not a major issue in this study.

### 5.2. Reliability and Validity

The results of the reliability and validity tests are reported in Table 3. The scales demonstrated strong internal consistency, with Cronbach’s α coefficients all exceeding the recommended threshold of 0.80. In terms of convergent validity, each item exhibited standardized factor loadings greater than 0.7, composite reliability values above 0.8, and average variance extracted values surpassing 0.5, indicating good convergent validity.

Structural validity was assessed through confirmatory factor analysis, as shown in Table 4. The nine-factor model demonstrated the best fit among all tested models, with all indices well within recommended thresholds (χ^2^/df = 1.087 < 3, RMSEA = 0.013 < 0.080, and GFI, CFI, NFI, TLI, and AGFI all exceeded 0.9).

Lastly, as presented in Table 5, the square root of the AVE for each construct was larger than its correlations with all other constructs, confirming good discriminant validity among constructs. We also calculate the HTMT values for all constructs following the recommended procedure. The results are presented in Table 6. All HTMT values are below the conservative threshold of 0.85 (Henseler et al., 2015), providing stronger evidence of discriminant validity among the study constructs again. Table 5 also indicates that item covariance is not a major concern. The overall fit is thus very strong, again supporting the validity of our model.

### 5.3. Main Effect Testing

Hierarchical regression analysis was conducted to test the proposed relationships between user experience, three dimensions of relational quality, and VCCB. This study employs a regression-based analytical approach rather than estimating the full structural model using SEM for the following two considerations. On the one hand, the study aims to examine differential effects of multiple dimensions of user experience and quality of relationship on VCCBs, and hierarchical regression allows for the estimation of incremental explanatory power of each dimension. On the other hand, PROCESS-based procedures provide a flexible and widely used approach for testing moderated mediation effects, particularly when using observed variables (Hayes, 2017). Therefore, the regression-based analysis is performed on validated constructs.

As shown in Table 7, In Model 1 (M1), the R^2^ is 0.269, with ΔR^2^ of 0.238, suggesting that adding the independent variables considerably improved the model’s explanatory power. Specifically, sensory experience (β = 0.112, *p* < 0.01), affective experience (β = 0.212, *p* < 0.001), intellectual experience (β = 0.239, *p* < 0.001), and behavioral experience (β = 0.157, *p* < 0.001) all positively predicted satisfaction, supporting H1a, H1b, H1c and H1d. Among the four user experience dimensions, intellectual experience showed the strongest effect on satisfaction (β = 0.239), followed by affective experience (β = 0.212) and behavioral experience (β = 0.157), with sensory experience having the smallest but still significant effect (β = 0.112). This pattern suggests that in virtual brand communities, cognitive stimulation (intellectual) and emotional bonding (affective) are relatively more important drivers of user satisfaction than sensory or behavioral cues. In Model 2 (M2), the R^2^ increased further to 0.290, higher than M1, with ΔR^2^ of 0.279, indicating that the inclusion of the independent variables significantly enhanced the explanatory power of the model. Sensory (β = 0.218, *p* < 0.001), affective (β = 0.161, *p* < 0.001), intellectual (β = 0.215, *p* < 0.001), and behavioral (β = 0.180, *p* < 0.001) experiences were all significant, thus supporting H1e, H1f, H1g, and H1h. In Model 3 (M3), the R^2^ was 0.266, with ΔR^2^ of 0.239, indicating improved explanatory power after adding the independent variables. Affective experience (β = 0.327, *p* < 0.001) and behavioral experience (β = 0.295, *p* < 0.001) emerged as significant predictors, supporting H1i and H1j. Notably, affective experience (β = 0.327) and behavioral experience (β = 0.295) emerged as the only significant predictors of commitment, whereas sensory and intellectual experiences were not significant. This differential pattern indicates that trust in virtual brand communities is built primarily through emotional connections and observable actions (e.g., active participation), rather than through sensory stimulation or intellectual exchange.

As shown in Model 5 (M5) Table 8, the R^2^ increased to 0.134 from 0.007 in M4, with ΔR^2^ of 0.127, indicating improved explanatory power. In Model 6 (M6), the R^2^ further increased to 0.259, significantly higher than in M4 and M5, with ΔR^2^ of 0.252 compared to M4, indicating superior explanatory power when adding the mediators. Satisfaction (β = 0.190, *p* < 0.001), trust (β = 0.197, *p* < 0.001), and commitment (β = 0.286, *p* < 0.001) were all significant predictors of VCCB, thus supporting H2a, H2b and H2c.

### 5.4. Mediating Effect

To further examine whether satisfaction, trust, and commitment mediate the relationship between four dimensions of user experience and VCCBs in VBCs, we followed Hayes’ (2017) recommendation and employed Model 4 of the PROCESS in SPSS 27.0. The bootstrap method with 5,000 resamples and a 95% confidence interval was used to test the mediating effects of satisfaction, trust, and commitment while controlling for the impact of demographic variables. The path analysis results are summarized in Table 9.

As shown in Table 9, satisfaction significantly mediated the effects of sensory experience (β = 0.087, 95% CI [0.056, 0.122]), affective experience (β = 0.115, 95% CI [0.076, 0.159]), intellectual experience (β = 0.119, 95% CI [0.079, 0.163]), and behavioral experience (β = 0.098, 95% CI [0.064, 0.135]) on VCCB. Therefore, H3a, H3b, H3c, and H3d were supported.

In addition, trust functioned as another significant mediator. The indirect effects of sensory experience (β = 0.115, 95% CI [0.078, 0.155]), affective experience (β = 0.117, 95% CI [0.076, 0.162]), intellectual experience (β = 0.123, 95% CI [0.083, 0.168]), and behavioral experience (β = 0.112, 95% CI [0.074, 0.155]) on VCCBs were all positive and significant. Thus, H3e, H3f, H3g, and H3h were supported.

Finally, commitment significantly mediated the effects of affective experience (β = 0.169, 95% CI [0.119, 0.221]) and behavioral experience (β = 0.143, 95% CI [0.097, 0.196]) on VCCBs. Hence, H3i and H3j were also supported.

### 5.5. Moderating Effect

To test the moderating role of community support, hierarchical regression analyses were conducted following the procedure recommended by Wen et al. (2005) in SPSS 27.0. To avoid multicollinearity, satisfaction, trust, commitment, and community support were mean-centered prior to constructing the interaction terms (satisfaction × community support, trust × community support, and commitment × community support).

**Figure 2:**
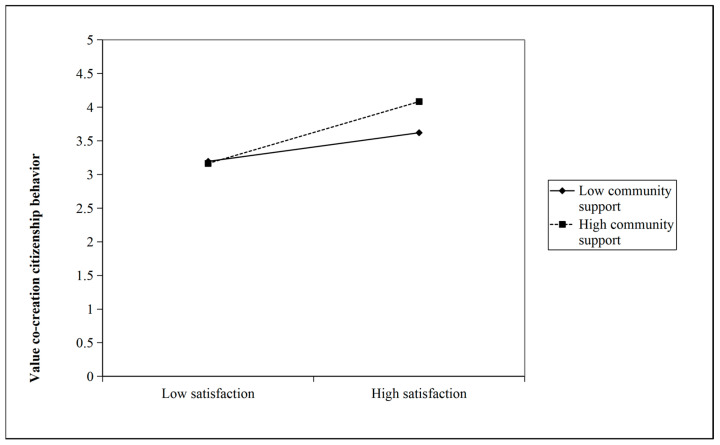
Moderating effect of community support on the relationship between satisfaction and VCCB.

As shown in Table 10, the interaction between satisfaction and community support had a significant positive effect on VCCBs (β = 0.127, *p* < 0.01), indicating that the effect of satisfaction on VCCBs varied significantly depending on the level of community support. Thus, H4a was supported. Figure 2 visualizes this moderating effect.

As shown in Table 11, the interaction between trust and community support was not significant (β = 0.049, *p* > 0.05), suggesting that community support did not moderate the effect of trust on VCCBs. Therefore, hypothesis H4b was not supported.

Similarly, Table 12 shows that the interaction between commitment and community support was also not significant (β = 0.055, *p* > 0.05), suggesting that community support did not moderate the effect of commitment on VCCBs. Therefore, H4c was not supported.

## 6. Discussions and Implications

### 6.1. Discussions

Grounded in relationship marketing theory and situated in the context of virtual brand communities (VBCs), this study develops and tests a comprehensive model to explain how multidimensional user experience is associated with value co-creation citizenship behaviors (VCCBs) through relational mechanisms and contextual conditions.

First, user experience is significantly and differentially associated with three dimensions of the quality of relationship. Specifically, intellectual experience is shown to have the strongest positive association with satisfaction (β = 0.239, *p* < 0.001), suggesting that cognitive stimulation and knowledge acquisition are central to users’ evaluative judgments of the community. Sensory experience is shown to have the strongest positive association with trust (β = 0.218, *p* < 0.001), highlighting the role of immersive and information-rich environments in reducing uncertainty and enhancing perceived reliability. Affective experience (β = 0.327, *p* < 0.001), followed by behavioral experience (β = 0.295, *p* < 0.001), is shown to have the strongest association with commitment, indicating that emotional resonance and sustained interaction are critical for fostering deeper relational attachment. These findings underscore the heterogeneous associations of user experience and distinct components of relationship.

Second, three dimensions of the quality of relationship are significantly positively associated with VCCBs, with commitment (β = 0.286) showing the strongest association, followed by trust (β = 0.197) and satisfaction (β = 0.190). This pattern suggests that while cognitive and evaluative states initiate engagement, deeper relational bonds are more strongly associated with sustaining discretionary, extra-role behaviors. High-quality relationships not only reduce perceived uncertainty but also foster emotional dependence and alignment with community goals, thereby motivating users to engage in value-enhancing activities.

Third, satisfaction, trust, and commitment serve as important and distinguished mediators linking user experience to VCCBs. Consistent with relationship marketing theory (Morgan & Hunt, 1994; Palmatier et al., 2006), experiential perceptions are internalized into relational evaluations, satisfaction, trust, and commitment, which subsequently helps explain behavioral engagement. Notably, commitment mediates the effects of affective and behavioral experiences more strongly, reflecting its deeper emotional foundation, whereas satisfaction and trust mediate the effects of all experiential dimensions.

Finally, community support plays a contingent rather than universal role in the relational process. Specifically, it significantly strengthens the relationship between satisfaction and value co-creation citizenship behaviors (VCCBs) but does not significantly moderate the effects of trust or commitment. This asymmetric pattern suggests that community support primarily amplifies users’ immediate evaluative responses, whereas deeper relational bonds, such as trust and commitment, are more strongly shaped by intrinsic motivations developed through repeated interactions over time (Garbarino & Johnson, 1999). Prior research distinguishes satisfaction as a transaction-specific and context-dependent evaluation, whereas trust and commitment represent more stable and enduring relational states that emerge through continuous relational exchanges (Garbarino & Johnson, 1999; Morgan & Hunt, 1994). As an external contextual resource, community support provides informational, emotional, and instrumental assistance that reinforces users’ positive evaluations of the community and activates reciprocity mechanisms (Eisenberger et al., 1986). Reciprocity theory further suggests that repeated reciprocal exchanges gradually foster trust, affective attachment, commitment, and relational solidarity among exchange partners (Molm, 2010). Accordingly, trust and commitment can be understood as deeply embedded relational outcomes accumulated through sustained social interaction, rather than immediate responses to contextual cues. Therefore, although community support can strengthen satisfaction-based reciprocity by enhancing users’ positive evaluative judgments, its incremental influence on the behavioral effects of trust and commitment is comparatively limited. This explains why the moderating effects of community support on the relationships between trust, commitment, and VCCBs are not statistically significant.

### 6.2. Theoretical Implications

This study makes several important contributions to literature. First, it enriches research on user experience by identifying its multidimensional nature and demonstrating its differentiated behavioral consequences. While prior studies often treat user experience as a unidimensional construct (Al-Shamaileh & Sutcliffe, 2023; Y. Chen et al., 2021; Y. Li et al., 2021; X. Wang & Zhou, 2023), this study shows that four dimensions of user experience—which are sensory, affective, intellectual, and behavioral experiences—exert distinct effects on three dimensions of relational quality including satisfaction, trust, and commitment. The pattern-mapping features of relationships revealed above provide important theoretical value for the understanding of how experiential components translate into relational outcomes. Moreover, by situating user experience within VBCs, a context characterized by continuous interaction and co-creation, this study extends existing research beyond commonly examined settings such as e-commerce and live streaming (Hsu, 2024; Pappas et al., 2019; Wani et al., 2017; Zhao & Wagner, 2024; H. Zhu & Chao, 2023).

Second, this study contributes to the literature on value co-creation by exploring the behavioral mechanism through which user experience influences VCCBs. Prior research has predominantly focused on direct effects or relied on social psychological explanations (Hasan et al., 2024; M. Yang et al., 2024). In contrast, this study introduces relationship marketing theory as an integrative framework and demonstrates that three dimensions of the quality of relationship serve as central mediators. This relational perspective provides a more process-oriented explanation of how experiential perceptions are transformed into sustained, citizenship-like behaviors, thereby enriching the theoretical foundation of value co-creation research.

Third, this study broadens the application of relationship marketing theory to digitally mediated community contexts. While RMT has been widely applied in traditional customer relationships and increasingly in digital marketing contexts (Achen, 2017; Afonso Vieira et al., 2011; Back et al., 2011; Chi et al., 2022; Jain et al., 2024; Steinhoff et al., 2019; Waters & Bortree, 2012), its application to user–community relationships in enterprise-led VBCs remains limited. By integrating experiential, relational, and contextual factors into a unified framework, this study demonstrates how RMT operates in interactive, user-driven environments and highlights the role of community support as a boundary condition. In doing so, it broadens the theoretical scope of RMT and enhances its relevance in the digital economy. That being said, it should be noted that the study contributes primarily by integrating established constructs into a new configuration within VBCs, rather than by introducing a wholly new theoretical logic.

### 6.3. Managerial Implications

This study also provides several actionable implications for practitioners managing VBCs. First of all, firms should adopt a multidimensional approach to design user experience. Rather than focusing solely on functional or informational aspects, managers should simultaneously enhance sensory, affective, intellectual, and behavioral experiences. This can be achieved by improving content quality and visual design, fostering emotionally engaging interactions, facilitating knowledge sharing, and enabling seamless user participation. For example, combining high-quality multimedia content with personalized and adaptive platform features can create immersive and adaptive user experiences that stimulate both cognitive and emotional engagement.

Second, firms should prioritize the development of high-quality relationships with users as a strategic lever for promoting value co-creation. Given the strong impact of commitment on VCCBs, managers should focus on fostering long-term relational bonds rather than short-term satisfaction. This can be achieved by establishing responsive service systems (e.g., integrating AI-based support with human interaction), offering personalized services (e.g., exclusive trials and tailored recommendations), and creating opportunities for meaningful user involvement (e.g., participatory decision-making and co-creation initiatives). Such practices enhance users’ sense of belonging and strengthen their willingness to contribute voluntarily.

Third, firms should strategically leverage community support mechanisms to enhance user engagement. The findings suggest that community support is particularly effective in amplifying the behavioral effects of satisfaction. Therefore, managers should invest in both instrumental and emotional support systems, such as knowledge-sharing platforms, peer assistance mechanisms, and recognition programs. For instance, implementing a tiered membership system that rewards user contributions, organizing community challenges, and offering symbolic incentives (e.g., badges and status recognition) can foster a supportive and participatory environment. By cultivating a positive community climate, firms can reinforce users’ favorable perceptions and encourage sustained participation in value co-creation activities.

Overall, this study highlights that effective management of VBCs requires a holistic approach that aligns experiential design, relational development, and supportive environments to stimulate users’ voluntary and value-enhancing behaviors.

## 7. Limitations and Future Research Directions

This study has several limitations that may limit the generalizability of the findings, but these limitations also open avenues for future research.

First, this study relies on self-reported measures to collect data, which may introduce potential common method bias. The future research could incorporate multi-source data including multiple survey sources, officially released objective data, and objective behavioral indicators, to further reduce this concern.

Second, the selection of control variables is primarily limited to user-level characteristics within VBCs, without accounting for factors at higher levels of analysis. Given that value co-creation behaviors are embedded in broader organizational and contextual environments, future research could incorporate multi-level controls, such as brand- or industry-level characteristics (e.g., industry type, brand maturity) and community-level attributes (e.g., community age, size, or governance structure). Incorporating such factors would enhance model robustness and provide a more comprehensive understanding of how contextual heterogeneity shapes user behavior.

Third, the sample composition presents limitations in terms of representativeness. The data are predominantly drawn from younger users, which may constrain the generalizability of the findings across broader groups. Given that age, digital literacy, and usage motivations may systematically influence both user experience and value co-creation behaviors, future studies should expand the sampling frame to include more diverse user populations across different age groups, cultural contexts, and usage patterns (Ma, 2006, 2007; Ma & Bu, 2021). Such efforts would improve external validity and enable intergroup comparisons. In addition, the sample is drawn from specific virtual brand communities within a single national context, which may limit the generalizability of the findings across different cultural and platform settings.

Fourth, while this study adopts relationship marketing theory to explain how user experience influences VCCBs, alternative theoretical mechanisms remain underexplored. Although the quality of relationship captures a key relational pathway, other mechanisms, such as structural embeddedness, social influence, or network position, may also shape users’ engagement in value co-creation. Future research could draw on complementary perspectives, such as social network theory, to examine how relational structures and interaction patterns influence citizenship behaviors in VBCs. Integrating multiple theoretical lenses would provide a more holistic understanding of value co-creation dynamics in digital communities.

Fifth, although a two-wave survey design was employed, the independent variable (user experience) and the mediating variables (satisfaction, trust, commitment) were measured at the same time. Therefore, this temporal separation limits the extent to which causal ordering of independent variables and mediating variables can be inferred. Future research could separate independent variables and mediating variables in data collection or adopt a three-wave longitudinal design which means collecting data for independent, mediating and dependent variables at different times so as to better establish causal relationships. More analysis could help better understand the findings by comparing the differences between respondents who completed both waves and those who did not. Future research can also employ SEM approach to better test the causal effects within the proposed framework.

## Figures and Tables

**Table 1:** Descriptive characteristics of respondents.

Variable	Category	Number	Percentage
Gender	Male	289	52.64%
	Female	260	47.36%
Age	Less than 18 years	22	4.01%
	18–30 years	257	46.81%
	31–40 years	146	26.59%
	41–60 years	104	18.94%
	60 years and over	20	3.64%
Education	High school and below	96	17.49%
	Junior college	106	19.31%
	Undergraduate	230	41.89%
	Graduate and above	117	21.31%
Length of participation	Less than one year	134	24.41%
	One to three years	199	36.25%
	Four to five years	141	25.68%
	More than five years	75	13.66%

**Table 2:** Measure Items.

Construct	Dimension	Item		Adapted From
User Experience	Sensory experience	SE1	This community makes a strong impression on my visual sense or other senses.	Brakus et al. (2009)
		SE2	I find it very interesting to see, hear, or interact with the brand in this community.	
		SE3	The community attracts me with its sensory (visual, auditory, etc.) appeal.	
	Affective experience	AE1	I feel emotionally uplifted when browsing the community.	
		AE2	The community generates positive emotions in me.	
		AE3	The community is a warm and emotional place.	
	Intellectual experience	IE1	Browsing the community stimulates positive and active thinking.	
		IE2	The community arouses my curiosity.	
		IE3	The community makes me think and solve problems.	
	Behavioral experience	BE1	When using the community, I want to engage in activities in it.	
		BE2	As a user of this community, I actively participate in it.	
		BE3	The community motivates my participation.	
Quality of Relationship	Satisfaction	RS1	I feel satisfied with the services provided by this community.	D. Li et al. (2006)
		RS2	The community meets my expectations.	
		RS3	The community makes me feel happy and delighted.	
	Trust	RT1	I trust the information and products provided by this community.	Doney and Cannon (1997)
		RT2	This community is reliable and trustworthy.	
		RT3	I believe the community does not take advantage of users.	
	Commitment	RC1	I am willing to maintain my relationship with this community.	Carlson et al. (2008)
		RC2	I am happy to comply with the community norms and participate in activities.	
		RC3	Even if it requires extra effort, I am willing to maintain my relationship with the community.	
Community Support		CS1	When I need help, the community helps me.	S. Wang et al. (2019)
		CS2	The community respects my values and goals.	
		CS3	The community values my input.	
		CS4	When I contribute to the community, I receive encouragement and support.	
Value Co-Creation Citizenship Behavior		BC1	I actively provide helpful suggestions to improve community services.	Yi and Gong (2013)
		BC2	I recommend the community to my friends.	
		BC3	I actively help other members in the community.	
		BC4	Even if the community makes errors or leaves me feeling disappointed during the service, I would remain tolerant.	

**Table 3:** Reliability and Convergent Validity.

Factor		Item	Standardized Factor Loading	CR	AVE	Cronbach’s α
User Experience	Sensory experience	SE1	0.877	0.886	0.723	0.886
		SE2	0.873			
		SE3	0.798			
	Affective experience	AE1	0.793	0.834	0.626	0.833
		AE2	0.753			
		AE3	0.826			
	Intellectual experience	IE1	0.794	0.831	0.622	0.831
		IE2	0.808			
		IE3	0.763			
	Behavioral experience	BE1	0.799	0.842	0.640	0.841
		BE2	0.83			
		BE3	0.769			
Quality of Relationship	Satisfaction	RS1	0.825	0.829	0.618	0.827
		RS2	0.8			
		RS3	0.731			
	Trust	RT1	0.801	0.846	0.646	0.846
		RT2	0.807			
		RT3	0.804			
	Commitment	RC1	0.805	0.844	0.643	0.843
		RC2	0.777			
		RC3	0.823			
Community Support		CS1	0.73	0.849	0.585	0.849
		CS2	0.754			
		CS3	0.798			
		CS4	0.776			
Value Co-Creation Citizenship Behavior		BC1	0.788	0.866	0.619	0.866
		BC2	0.755			
		BC3	0.825			
		BC4	0.777			

**Table 4:** Structural Validity.

Model	χ^2^/df	RMSEA	GFI	CFI	NFI	TLI	AGFI
Nine-factor model	1.087	0.013	0.956	0.996	0.954	0.995	0.944
Eight-factor model	3.575	0.069	0.843	0.884	0.847	0.865	0.804
Seven-factor model	3.753	0.071	0.834	0.873	0.836	0.855	0.797
Six-factor model	5.612	0.092	0.763	0.784	0.750	0.758	0.716
Five-factor model	8.601	0.118	0.659	0.639	0.612	0.601	0.596
Four-factor model	8.062	0.114	0.683	0.661	0.632	0.629	0.629
Three-factor model	8.454	0.117	0.684	0.639	0.611	0.609	0.633
Two-factor model	11.312	0.137	0.6	0.498	0.477	0.458	0.537
Single-factor model	12.537	0.145	0.58	0.437	0.419	0.394	0.515

Notes: SE = sensory experience; AE = affective experience; IE = intellectual experience; BE = behavioral experience; RS = satisfaction; RT = trust; RC = commitment; CS = community support; BC = value co-creation citizenship behavior. The nine-factor model includes SE, AE, IE, BE, RS, RT, RC, CS, and BC. The eight-factor model includes SE, AE, IE, BE, RS, RT, RC, and CS + BC. The seven-factor model includes SE, AE, IE, BE, RS + RT + RC, CS, and BC. The six-factor model includes SE + AE + IE + BE, RS, RT, RC, CS, and BC. The five-factor model includes SE + AE + IE + BE, RS, RT, RC, and CS + BC. The four-factor model includes SE + AE + IE + BE, RS + RT + RC, CS, and BC. The three-factor model includes SE + AE + IE + BE + RS + RT + RC, CS, and BC. The two-factor model includes SE + AE + IE + BE + RS + RT + RC and CS + BC. The single-factor model is SE + AE + IE + BE + RS + RT + RC + CS + BC.

**Table 5:** Discriminative validity test.

	1	2	3	4	5	6	7	8	9
1. Sensory experience	0.850								
2. Affective experience	0.423 **	0.791							
3. Intellectual experience	0.252 **	0.188 **	0.789						
4. Behavioral experience	0.334 **	0.260 **	0.308 **	0.800					
5. Satisfaction	0.317 **	0.353 **	0.367 **	0.322 **	0.786				
6. Trust	0.403 **	0.342 **	0.359 **	0.361 **	0.352 **	0.804			
7. Commitment	0.433 **	0.416 **	0.354 **	0.384 **	0.346 **	0.361 **	0.802		
8. Community Support	0.084 *	0.090 **	0.129 **	0.052	0.115 **	0.132 **	0.047	0.765	
9. VCCB	0.240 **	0.224 **	0.209 **	0.300 **	0.350 **	0.366 **	0.420 **	0.136 **	0.787
AVE	0.723	0.626	0.622	0.640	0.618	0.646	0.643	0.585	0.619

Note: * *p* < 0.05; ** *p* < 0.01.

**Table 6:** The result of the HTMT test.

	1	2	3	4	5	6	7	8	9
1. Sensory experience									
2. Affective experience	0.497								
3. Intellectual experience	0.294	0.228							
4. Behavioral experience	0.392	0.317	0.374						
5. Satisfaction	0.371	0.430	0.445	0.388					
6. Trust	0.468	0.411	0.426	0.426	0.421				
7. Commitment	0.504	0.496	0.424	0.459	0.416	0.425			
8. Community Support	0.098	0.105	0.155	0.062	0.135	0.157	0.057		
9. VCCB	0.277	0.263	0.247	0.357	0.413	0.425	0.489	0.158	

**Table 7:** Regression Analysis of User Experience on the Quality of Relationship.

Variable		Satisfaction		Trust		Commitment	
		M1		M2		M3	
		β	t	β	t	β	t
Control Variables	Gender	0.048	1.288	0.003	0.092	−0.009	−0.234
	Age	−0.010	−0.271	−0.05	−1.353	−0.040	−1.072
	Education	−0.024	−0.649	0.017	0.458	0.054	1.453
	Length of participation	−0.112	−2.977	−0.002	−0.057	−0.071	−1.886
Independent Variables	Sensory experience	0.112 **	2.633	0.218 ***	5.218	-	-
	Affective experience	0.212 ***	5.116	0.161 ***	3.951	0.327 ***	8.497
	Intellectual experience	0.239 ***	6.050	0.215 ***	5.530	-	-
	Behavioral experience	0.157 ***	3.849	0.180 ***	4.502	0.295 ***	7.726
R^2^		0.269		0.290		0.266	
ΔR^2^		0.238		0.279		0.239	
F		24.882		27.555		32.671	

Note: ** *p* < 0.01, *** *p* < 0.001.

**Table 8:** Regression Analysis of VCCBs on User Experience and Quality of Relationship.

Variable		VCCBs					
		M4		M5		M6	
		β	t	β	t	β	t
Control Variables	Gender	0.000	0.001	0.006	0.161	0.002	0.049
	Age	−0.022	−0.512	−0.033	−0.814	−0.010	−0.253
	Education	0.070	1.621	0.031	0.766	0.020	0.536
	Length of participation	−0.027	−0.634	0.005	0.114	0.049	1.296
Independent Variables	Sensory experience			0.097 *	2.093		
	Affective experience			0.109 *	2.417		
	Intellectual experience			0.098 *	2.277		
	Behavioral experience			0.208 ***	4.698		
Mediator variables	Satisfaction					0.190 ***	4.587
	Trust					0.197 ***	4.777
	Commitment					0.286 ***	6.930
R^2^		0.007		0.134		0.259	
ΔR^2^		0.007		0.127		0.252	
F		0.890		10.411		26.988	

Note: * *p* < 0.05, *** *p* < 0.001.

**Table 9:** Results on Indirect effect for different paths.

Path	Effect Value	Standard Error	Bootstrap 95%CI	
			Lower	Upper
Sensory experience → Satisfaction → VCCBs	0.087	0.017	0.056	0.122
Affective experience → Satisfaction → VCCBs	0.115	0.021	0.076	0.159
Intellectual experience → Satisfaction → VCCBs	0.119	0.022	0.079	0.163
Behavioral experience → Satisfaction → VCCBs	0.098	0.019	0.064	0.135
Sensory experience → Trust → VCCBs	0.115	0.020	0.078	0.155
Affective experience → Trust → VCCBs	0.117	0.022	0.076	0.162
Intellectual experience → Trust → VCCBs	0.123	0.022	0.083	0.168
Behavioral experience → Trust → VCCBs	0.112	0.021	0.074	0.155
Affective experience → Commitment → VCCBs	0.169	0.026	0.119	0.221
Behavioral experience → Commitment → VCCBs	0.143	0.025	0.097	0.196

Note: “→” indicates the indirect effect path among variables.

**Table 10:** Moderating Effect of Community Support on the Impact of Satisfaction on VCCBs.

Variable		VCCBs					
		M7		M8		M9	
		β	t	β	t	β	t
Control Variables	Gender	0.000	0.001	−0.012	−0.296	−0.017	−0.438
	Age	−0.022	−0.512	−0.019	−0.469	−0.020	−0.507
	Education	0.070	1.621	0.058	1.441	0.064	1.607
	Length of participation	−0.027	−0.634	0.031	0.765	0.027	0.659
Mediator variable	Satisfaction			0.344 ***	8.417	0.346 ***	8.547
Moderator variable	Community support			0.095 *	2.351	0.112 **	2.779
Interaction term	Satisfaction × Community support					0.127 **	3.164
R^2^		0.007		0.137		0.153	
Adjusted R^2^		−0.001		0.128		0.142	
ΔR^2^		0.007		0.009		0.016	
F		0.890		14.349		13.934	

Note: * *p* < 0.05, ** *p* < 0.01, *** *p* < 0.001.

**Table 11:** Moderating Effect of Community Support on the Impact of Trust on VCCBs.

Variable		VCCBs					
		M10		M11		M12	
		β	t	β	t	β	t
Control Variables	Gender	0.000	0.001	0.003	0.073	0.000	−0.005
	Age	−0.022	−0.512	−0.005	−0.123	−0.003	−0.066
	Education	0.070	1.621	0.042	1.039	0.043	1.077
	Length of participation	−0.027	−0.634	−0.007	−0.171	−0.009	−0.222
Mediator variable	Trust			0.350 ***	8.694	0.354 ***	8.757
Moderator variable	Community support			0.089 *	2.212	0.095 *	2.353
Interaction term	Trust × Community support					0.049	1.214
R^2^		0.007		0.144		0.146	
Adjusted R^2^		−0.001		0.134		0.135	
ΔR^2^		0.007		0.008		0.002	
F		0.890		15.157		13.214	

Note: * *p* < 0.05, *** *p* < 0.001.

**Table 12:** Moderating Effect of Community Support on the Impact of Commitment on VCCBs.

Variable		VCCBs					
		M13		M14		M15	
		β	t	β	t	β	t
Control Variables	Gender	0.000	0.001	0.016	0.406	0.012	0.308
	Age	−0.022	−0.512	−0.011	−0.278	−0.009	−0.232
	Education	0.070	1.621	0.023	0.588	0.023	0.602
	Length of participation	−0.027	−0.634	0.021	0.542	0.020	0.513
Mediator variable	Commitment			0.414 ***	10.571	0.418 ***	10.658
Moderator variable	Community support			0.116 **	3.003	0.120 **	3.092
Interaction term	Commitment × Community support					0.055	1.407
R^2^		0.007		0.191		0.194	
Adjusted R^2^		−0.001		0.182		0.184	
ΔR^2^		0.007		0.013		0.003	
F		0.890		21.336		18.604	

Note: ** *p* < 0.01, *** *p* < 0.001.

## Data Availability

The data are available from the corresponding author upon request.
